# Nucleoporin 62-Like Protein Is Required for the Development of Pharyngeal Arches through Regulation of Wnt/β-Catenin Signaling and Apoptotic Homeostasis in Zebrafish

**DOI:** 10.3390/cells8091038

**Published:** 2019-09-05

**Authors:** Xiaojie Yang, Xixi Li, Qilin Gu, Qing Li, Zongbin Cui

**Affiliations:** State Key Laboratory of Freshwater Ecology and Biotechnology, Institute of Hydrobiology, Chinese Academy of Sciences, Wuhan 430072, China

**Keywords:** Nup62l, craniofacial development, pharyngeal arches, Wnt/β-catenin signaling, apoptosis

## Abstract

We have previously observed the predominant expression of nucleoporin 62-like (Nup62l) mRNA in the pharyngeal region of zebrafish, which raises the question whether Nup62l has important implications in governing the morphogenesis of pharyngeal arches (PA) in zebrafish. Herein, we explored the functions of Nup62l in PA development. The disruption of Nup62l with a CRISPR/Cas9-dependent gene knockout approach led to defective PA, which was characterized by a thinned and shortened pharyngeal region and a significant loss of pharyngeal cartilages. During pharyngeal cartilage formation, prechondrogenic condensation and chondrogenic differentiation were disrupted in homozygous *nup62l*-mutants, while the specification and migration of cranial neural crest cells (CNCCs) were unaffected. Mechanistically, the impaired PA region of *nup62l*-mutants underwent extensive apoptosis, which was mainly dependent on activation of p53-dependent apoptotic pathway. Moreover, aberrant activation of a series of apoptotic pathways in *nup62l*-mutants is closely associated with the inactivation of Wnt/β-catenin signaling. Thus, these findings suggest that the regulation of Wnt/β-catenin activity by Nup62l is crucial for PA formation in zebrafish.

## 1. Introduction

Craniofacial abnormalities are common congenital defects and usually result from the aberrant development of pharyngeal arches (PA) [1]. The craniofacial morphogenesis of vertebrates is a complex and precise process involving neuromuscular and musculoskeletal connections. Neural crest cells (NCCs), representing a highly specialized pluripotent cell population of vertebrates, stem from the dorsal neural tube and can generate a diverse array of adult tissue types [2]. Pharyngeal skeletal elements of vertebrates originate from NCCs and the paraxial mesoderm [3]. Specifically, craniofacial neural crest cells (CNCCs) arising from the midbrain and hindbrain of embryos can migrate to the PA region and form cartilage, pigment cells, connective tissues, and sensory neurons while the surrounding paraxial mesoderm gives rise to pharyngeal muscles and endothelia [4,5].

During zebrafish embryogenesis, the cartilaginous craniofacial skeleton is mainly composed of seven PA, which is a paired structure supporting the feeding and gill-breathing structures, and a dorsal neurocranium that protects brain and sense organs, and develops in a stereotyped pattern [6]. In zebrafish larvae, the seven paired PA arise on each side of the midline and contain distinct dorsal and ventral sets of cartilage [3]. CNCCs migrate ventrolaterally in three distinct streams (mandibular, hyoid, and branchial) to populate seven pharyngeal segments, form distinctive condensations of precartilage mesenchyme [7,8], and commence their subsequent chondrification [6]. Eventually, CNCC-derived cartilages in the first PA (mandibular) aid in the U-shaped jaw formation and cartilages in the second PA (hyoid) essentially serve to support the jaw structure, while those within the more posterior five PA contribute to the branchial gill structures [9]. 

The advantages of high fecundity, transparent embryos, and small size make zebrafish a powerful model for specialized mutagenesis screens for the identification of genes whose counterparts can regulate craniofacial development in humans [10]. For example, both the ventral arch defects in zebrafish mutations *suc/et1* and cartilage fusions in *vgo* mutants, as well as the phenotypes of *et1*^-/-^ mice, resemble the phenotypes observed in humans suffering from DiGeorge and velocardiofacial syndromes [11,12]. The combination of pigment and enteric neuronal defects observed in zebrafish *cls/sox10* mutants [13], is reminiscent of the human Waardenburg–Shah syndrome and Hirschsprung’s disease, which are characterized by malformed otic region and pigmentation [14]. The morphologic anomalies of *yot/gli2* mutants resemble holoprosencephaly in human with mutations in Shh signaling, which are characterized by defective midfacial skeletal and ventral brain [15]. Therefore, the mechanistic dissection of PA development in zebrafish may provide novel targets for therapies of human craniofacial syndromes and repair.

PA formation is an exquisitely controlled and evolutionarily conserved process across vertebrates [10]. Each PA is composed of a mesodermal core surrounded by CNCCs that is encapsulated by ectoderm and endoderm [16]. The complex interactions between these different embryonic cell types are crucial for establishing signaling networks to govern the proper PA patterning [17]. For example, Fgf and Bmp signaling co-regulate the jaw joint development by regulating the expression of downstream factors both in chick and zebrafish [18,19]. Deprivation of endothelin-1 (Edn1) or disruption of its signaling pathway cause severe jaw truncations in murine and zebrafish embryos [2]. In zebrafish, Bmp also functions upstream and in parallel to Edn1 to coordinate ventral patterning of PA [20]. Moreover, Edn1 signaling and Jagged-Notch signaling can act antagonistically to subdivide dorsoventral (DV) domains within the arches [21].

Wnt signaling is intimately involved in craniofacial morphogenesis. Dysregulation of Wnt activity leads to facial anomalies. WNT3 mutations in human and genetic disruption of Wnt9b in mice have been closely implicated in cleft lip/palate [22,23]. In mice, conditional inactivation of β-catenin with Wnt1-Cre leads to defective formation of craniofacial structures derived from NCCs [24]. Lrp6 deprivation causes a cleft lip with cleft palate in mice and Lrp6-mediated Wnt/β-catenin signaling controls the formation and fusion of facial primordia [25]. At different developmental stages in murine embryogenesis, Wnt/β-catenin signaling can not only facilitate chondrogenesis through potentiating migration and differentiation of NCCs, but also exert inhibitory effects on chondrogenesis through inducing the transcriptional repressor Twist1 that restricts BMP2-induced expression of chondrocyte genes [24,26]. In zebrafish, Wnt4a and Wnt11r, which stem from the head mesoderm and ectoderm, respectively, are actively involved in the segmental formation of pharyngeal pouches [27]. Moreover, Wnt9a, Wnt11 and Wnt5b are indispensable for the development of chondrocytes and PA [28,29,30]. Nevertheless, the functional mechanisms of Wnt/β-catenin signaling during PA formation in zebrafish remain largely unknown.

Several investigations convey novel and important evidence to the essential requirement of certain nucleoporins (Nups) in PA development. Loss of *elys/flo* function in zebrafish confers phenotypic malformations in PA, retina and intestine with massive apoptosis in these disturbed tissues [31]. Zebrafish *nup107*-mutants display a disappearance of PA and severe impairment of the eyes and intestine due to the perturbation of nuclear pore function [32]. Nup62 is one of the core molecular components of the nuclear pore complexes (NPC) and localizes at the central channel of NPC [33]. Nup62 is predominantly implicated in selective nucleocytoplasmic transport events and regulation of nuclear pore permeability [34,35]. Intriguingly, sporadic clues support the involvement of Nup62 in human craniofacial disorders such as human autosomal recessive infantile bilateral striatal necrosis (degeneration of the basal ganglia) [36]. We have unraveled the crucial roles of Nup62l in developmental programs during zebrafish embryogenesis, including DV patterning, gastrula convergence and extension movements and specification of midline organ precursors. Importantly, the predominant expression of *nup62l* in PA region suggests its potential role in regulating PA morphogenesis [37]. These compelling cues prompted us to assess whether Nup62l was obligatory for proper formation of PA in zebrafish. 

In this study, we addressed an in vivo role of Nup62l in PA development of zebrafish. Deletion of *nup62l* with CRISPR/Cas9-mediated approach led to morphological anomalies in pharynx and severe loss of cartilages in the PA due to impaired condensation and the differentiation of pharyngeal chondrogenic progenitors. Further, we found that extensive apoptosis occurred within the defective PA due to the activation of multiple intrinsic and extrinsic apoptotic pathways, especially the p53-dependent apoptotic pathway. Moreover, we demonstrated that the aberrant activation of these apoptotic pathways was closely associated with the suppression of Wnt/β-catenin signaling in *nup62l*-mutants. 

## 2. Materials and Methods

### 2.1. Zebrafish Husbandry

Zebrafish AB line and mutants were maintained in our laboratory as described [37]. Embryos were collected from natural spawning and incubated at 28 °C and staged according to standard protocols [38]. All experiments were performed following animal use protocols approved by the Animal Care and Use Committee of the Institute of Hydrobiology, Chinese Academy of Sciences (approval ID Keshuizhuan 0829).

### 2.2. CRISPR/Cas9-Mediated Gene Knockout

The target sequence for *nup62l* was 5’-GGGGCTTCAACCACTGGGACAGG-3’, which was designed using an online tool ZiFiT Targeter software (http://zifit.partners.org/ZiFiT). The CRISPR/Cas9-mediated genome editing approach in zebrafish was conducted as previously described [39].

### 2.3. Constructs, Antisense Morpholino (MO), mRNA Microinjection and Chemical Treatments

Constructs used to synthesize riboprobes for in situ hybridization were generated by subcloning DNA fragment of target genes into pBluescript II ks (+) vector. The *p53*-antisense morpholino (*p53*-MO) targeting the start codon (5’-GCGCCATTGCTTTGCAAGAATTG-3’) was purchased from Gene Tools [40]. The construct for *in vitro* synthesis of zebrafish *nup62l* mRNA was prepared as described previously [37]. We microinjected one-cell stage embryos with an optimal concentration of 5 ng MO/embryo, or an optimal dosage of 300 pg mRNA/embryo. The *nup62l*^-/-^ embryos were treated with 10 μM Wnt agonist 1, and wild-type (WT) embryos injected without or with 300 pg *nup62l* mRNA were treated with either 15 μM XAV939 or 0.15% DMSO at 6 hpf and processed for TUNEL assays at 72 hpf.

### 2.4. Quantitative Real-Time PCR (qRT–PCR)

Total RNAs were prepared from 50 embryos at the indicated stages using Trizol reagent (Invitrogen, Carlsbad, CA, USA) according to the manufacturer’s protocol. A first strand cDNA synthesis kit (Thermo Scientific, Waltham, MA, USA) was used to synthesize cDNAs and qRT–PCR was carried out as described previously [41]. Primer sequences for *sox9a*, *col2a1a*, *sox9b*, *caspase 8*, *caspase 9*, *fadd*, *tp53*/*p53*, *mdm2*, *bbc3*, *gadd45al*, *fsta*, *tp53bp1* and *tp53i11a/b* were listed in Table 1.

### 2.5. Whole-Mount In Situ Hybridization (WISH)

Zebrfish embryos at desired stages were fixed in 4% paraformaldehyde (PFA) overnight before processing for WISH analysis as described [37]. Digoxigenin-UTP-labeled antisense RNA probes for *sox9a*, *sox10*, *foxd3*, *dlx2a*, *col2a1a*, *caspase 3a*, *caspase 3b*, *caspase 8*, *caspase 9*, *tp53*, *mdm2*, *bbc3*, *fsta* and *gadd45al* were generated with an in vitro transcription method using T7 or T3 RNA polymerase. Embryos were imaged using a SteReo Lumar V12 microscope from Carl Zeiss (ZEISS, Oberkochen, Germany).

### 2.6. Alcian Blue Staining

Alcian blue staining of cartilages was carried out as described previously with minor modifications [9,42]. Briefly, zebrafish larvae were collected at 96 hpf, fixed in 4% PFA overnight, and treated with alcian blue solution (0.1%) dissolved in 80% ethanol/20% glacial acetic acid overnight. Specimens were subjected to several washes of acid alcohol and then transferred into 1% potassium hydroxide/3% hydrogen peroxide solution to further clear pigmentation. Subsequently, embryonic tissues were digested in 0.05% trypsin for two hours. Cartilage preparations were mounted in 70% glycerol and visualized on a SteReo Lumar V12 microscope from Carl Zeiss.

### 2.7. TUNEL Assay and Detection of Caspase 3 Activity

Apoptosis were detected with TUNEL assay. Embryos were harvested at the indicated stages, fixed in 4% PFA, incubated in 1μg/ml Proteinase K solution to solubilize tissues and rinsed twice with PBS-Tween. Apoptotic cells were labeled using ApopTag® Red In Situ Apoptosis Detection Kit (Millipore, Billerica, MA, USA) following the manufacture’s instruction. Fluorescent images were taken with a LSM 710 confocal microscope from Carl Zeiss. To detect the activity of caspase 3 in zebrafish, embryos were collected at 60 hpf and homogenated in ice-cold RIPA buffer. Then, the homogenate was mixed with an equal volume of 0.39 mM of Acetyl-Asp-Glu-Val-Asp-7-amido-4-methylcoumarin (Sigma-Aldrich, Saint Louis, MO, USA), a fluorogenic substrate for caspase 3. Quantification of caspase 3 activity was performed by fluorescent detection of free AMC (also known as 7-amino-4-methylcoumarin) as described previously [43].

## 3. Results

### 3.1. Nup62l Depletion Impaired the Development of PA

**Figure 1:**
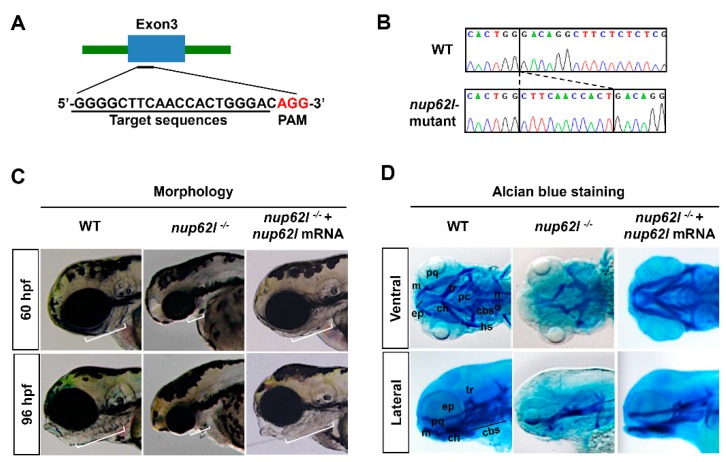
Loss of Nup62l led to severely impaired formation of PA cartilages. (**A)** Diagram showing the CRISRP/Cas9 target DNA sequence (underline) of zebrafish gene *nup62l*. PAM region (AGG) was shown in red. (**B**) Sanger sequencing results revealed an 11-bp genomic DNA fragment insertion at the target site in *nup62l*-mutants. (**C**) Lateral views showing the pharyngeal morphology of WT siblings. Embryos of *nup62l*-mutants at 60 hpf or 96 hpf were injected with or without 300 pg *nup62l* mRNA/embryo. White lines indicated the pharyngeal regions. Note a shortened and thinned pharyngeal region in *nup62l*-mutants. (**D**) Images showing pharyngeal cartilage elements stained with alcian blue in WT. The *nup62l*-mutants injected with or without 300 pg *nup62l* mRNA at 96 hpf were ventrally viewed in upper panels and laterally viewed in lower panels. cbs, ceratobranchials; ch, ceratohyal; ep, ethmoid plate; hs, hyosymplectic; m, Meckel’s cartilage; no, notochord; pc, parachordal; pq, palatoquadrate; tr, trabecula.

We have previously shown that *nup62l* was expressed ubiquitously at early stages and the strongest signals were enriched in some specific organs/tissues at posterior stages, particularly in PA region [37]. Therefore, it is reasonable to speculate that Nup62l may be involved in the development of zebrafish PA. To verify the hypothesis, we employed CRISPR/Cas9 system to generate homozygous *nup62l*-knockout mutants (*nup62l^-/-^)* by targeting the third exon of *nup62l* (Figure 1A). DNA sequencing results indicated that homozygous *nup62l*-mutants harbored an 11-bp insertion within genomic DNA at the target site (Figure 1B; Supplementary Figure S1A), leading to a frame-shift mutation of open reading frame and a premature stop codon that can abolish all functions of Nup62l (Figure S1B). We observed the phenotypic defects of PA caused by Nup62l deprivation. At 60 and 96 hpf, *nup62l*-mutants were distinguishable from wild-type (WT) siblings through the severely impaired PA showing concomitant thinned and shortened pharyngeal region (Figure 1C; Supplementary Figure S1C). To further characterize PA defects, we compared the pharyngeal cartilage patterns in *nup62l*-mutants with those in WT after staining with alcian blue, a dye used for detecting the extracellular matrix associated with chondrocytes [44]. By 96 hpf, about 89% of *nup62l*-mutants showed a complete loss of cartilages in all of seven PA, but cartilaginous neurocranium was not significantly affected (Figure 1D; Supplementary Figure S1D–F). In addition, palatoquadrate (pq) and Meckel’s (m) cartilages of the PA1, hyosymplectic (hs) and ceratohyal (ch) cartilages of the 2nd PA, as well as ceratobranchials (cb) and hypobranchials (hb) cartilages of the more posterior 3rd–7th PA were strikingly missing, whereas the length and width of the cartilaginous neurocranium appeared unperturbed (Figure 1D; Supplementary Figure S1E,F). We found that forced expression of *nup62l* mRNA rescued defective PA to normal (Figure 1C,D; Supplementary Figure S1C–F). In addition, the N-terminal 381bp (127AA) length partial mRNA of *nup62l* coding sequences did not have a dominant-negative effect since WT embryos injected with this short mRNA fragment exhibited normal development of PA (Figure S1G), suggesting that the abnormal PA phenotype of homozygous *nup62l*-mutants was indeed caused by CRISPR/Cas9-mediated *nup62l* knockout. These data suggest a crucial role of Nup62l in PA development during zebrafish embryogenesis.

### 3.2. Nup62l Loss-of-Function Disrupted Precondrogenic Condensation and Chondrogenic Differentiation during Pharyngeal Cartilage Formation

**Figure 2:**
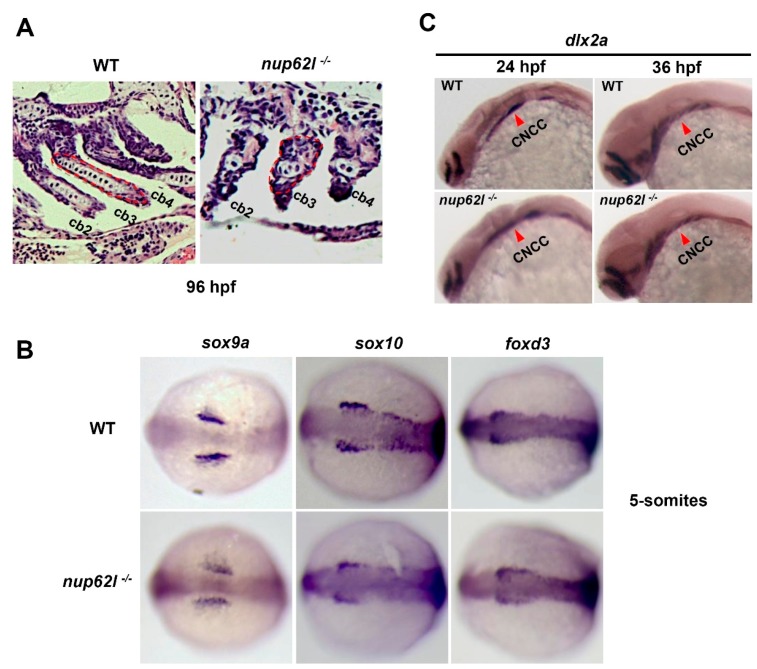
Specification and migration of CNCCs were unperturbed by loss of Nup62l. (**A**) Representative images showing the severely impaired PA chondrocytes of *nup62l*-mutants on sections stained with hematoxylin and eosin at 96 hpf. The 2nd–4th ceratobranchials (cb) were indicated. (**B**) Dorsal views with anterior region toward the left of 5-somites embryos stained with early crest markers (*sox9a*, *sox10* and *foxd3*). (**C**) WT and *nup62l*-mutant embryos were stained with probes of *dlx2a*, a marker of CNCCs migration at 24 hpf and 36 hpf. Lateral views of CNCCs as pointed by red arrowheads.

The cartilages of PA were reported to derive from CNCCs in zebrafish [45]. The formation of pharyngeal cartilages involves multiple steps, including specification of CNCCs, migration of CNCCs into seven pharyngeal segments, mesenchymal condensation formation and chondrocytes differentiation [3,6]. We first assessed the chondrocytes in PA through staining *nup62l*-mutants and WT siblings at 96 hpf with hematoxylin and eosin (H&E) followed by sagittal sections analysis. A large number of well-ordered chondrocytes were arranged normally within the arches of WT larvae, while a few of disorganized chondrocytes were noticed in impaired arches of *nup62l*-mutants (Figure 2A; Supplementary Figure S2A). To further identify the disrupted steps of cartilage formation by Nup62l deprivation, the expression of corresponding markers were detected with WISH. In *nup62l*-mutants, expression of the early crest markers *sox9a*, *sox10* and *foxd3* at 5-somites stage was unperturbed in comparison with that in WT controls (Figure 2B; Supplementary Figure S2B), indicating the specification of CNCCs occurred appropriately. Ventrolateral migration of CNCCs to PA was monitored by detecting the postmigratory neural crest marker *dlx2a* [46]. The expression of *dlx2a* was indistinguishable in WT and *nup62l*-mutants at 24 and 36 hpf (Figure 2C; Supplementary Figure S2C), suggesting an unaffected migratory behavior of CNCCs in *nup62l*-mutants. 

**Figure 3:**
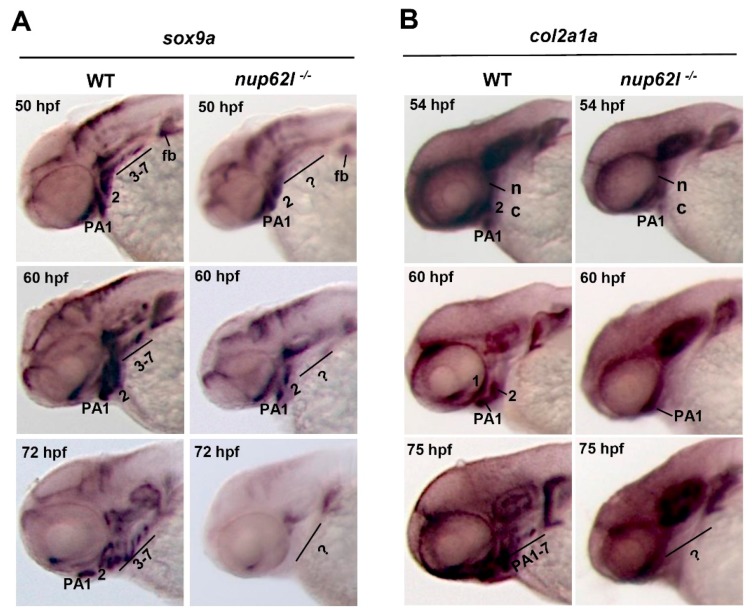
Nup62l was essential for condensation and differentiation of pharyngeal chondrogenic progenitors. WISH expression patterns of markers *sox9a* (**A**) and *col2a1a* (**B**) in *nup62l*-mutants at indicated stages. Embryos in (**A**) and (**B**) were shown as lateral views with anterior to the left. Question markers indicate the pharyngeal regions with no or unrecognized PA. PA, pharyngeal arches; fb, fin bud; nc, neurocranium.

Sox9a is a transcription factor that is obligatory for chondrogenesis [47] and expressed in CNCC mesenchymal condensations in PA [48,49], and Col2a1a, a predominant extracellular matrix protein in cartilage, marks differentiating chondrocytes in PA [50,51]. A significantly attenuated expression of *sox9a* in PA 3-7 and a slightly reduced expression in ventral parts of the first two arches were detected in *nup62l*-mutants at 50 hpf. At 60 hpf, *sox9a* transcripts at reduced levels appeared in condensations within the first and second arches. However, the expression of *sox9a* was completely lost in condensations within the entire PA at 72 hpf (Figure 3A; Supplementary Figure S3A). Consistent with the fact that *col2a1a* expression is dependent on *sox9a* in CNCCs [52], *nup62l*-mutants exhibited an obvious reduction of *col2a1a* expression in PA at 54 and 60 hpf, and a complete absence of seven PA at 75 hpf (Figure 3B; Supplementary Figure S3B). Consistently, qRT–PCR analysis, used to examine the expression of *sox9a*, *col2a1a* and *sox9b* in *nup62l*-mutants and WT embryos, showed that mRNA levels of *sox9a and col2a1a* decreased in *nup62l*-mutants. However, the expression of *sox9b* seemed unaffected in *nup62l*^-/-^ embryos when compared with that in WT (Figure S3C). These data indicate that loss of Nup62l interrupts the condensation and differentiation of pharyngeal chondrogenic progenitors, and thus leads to the hypoplastic PA in zebrafish.

### 3.3. Extensive Apoptosis Occurred in the Defective PA of Nup62l-Mutants

**Figure 4:**
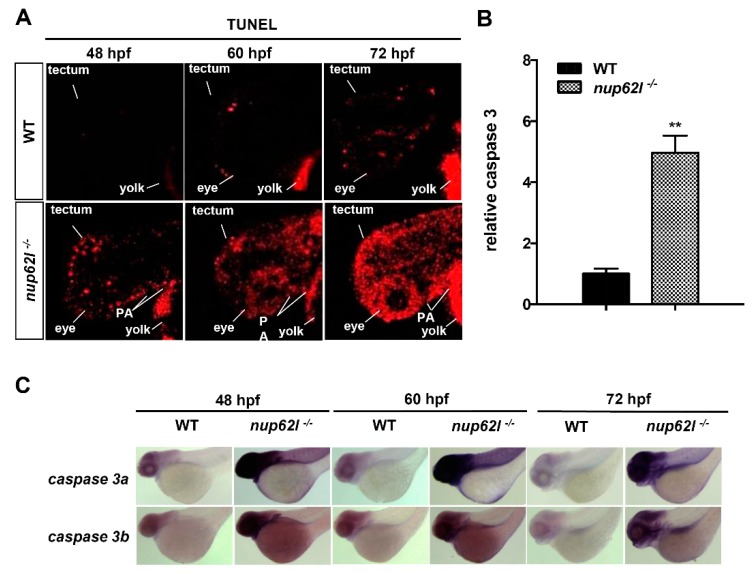
Loss of Nup62l induced extensive apoptosis in the impaired PA region. (**A**) Detection of apoptotic cells in PA of *nup62l*-mutants with TUNEL assays at indicated stages. PA, pharyngeal arches. (**B**) Strongly elevated caspase 3 activity in *nup62l*-mutants at 60 hpf in comparison with that in WT siblings. The data expressed as Mean ± SD were representatives of three independent experiments, each done with three samples of 15 zebrafish. ** *p* < 0.01. (**C**) WISH assays showing lateral views of the expression of *caspase 3a* and *caspase 3b* genes in WT or *nup62l*-mutants at 48 hpf, 60 hpf or 72 hpf.

Severely defective PA and reduced chondrocytes within pharyngeal segments of *nup62l*-mutants suggested that abnormal apoptosis might occur during the formation of pharyngeal cartilage. We thereby assessed the status of apoptotic cells in *nup62l*-mutants and controls at different developmental stages utilizing TUNEL assays. Apoptotic cells were almost undetectable at 48 hpf, and only few and sporadic dead cells could be observed at 60 and 72 hpf in normally developing WT. However, *nup62l*-mutants from 48 hpf onward showed a progressively augmented number of apoptotic cells, which are predominantly distributed throughout PA, eyes and optic tectum (Figure 4A; Supplementary Figure S4A,B).

Caspase 3, known as the executioner of apoptosis, can be activated by both extrinsic (death receptor-mediated caspase 8 signaling) and intrinsic (mitochondria-dependent caspase 9 signaling) apoptotic pathways [53]. Since activation of caspase 3 is a common essential step in the apoptotic process, we first detected the activation levels of it. As shown in Figure 4B, the activity of caspase 3 in *nup62l*-mutants was up-regulated approximately five-fold compared with WT siblings at 60 hpf. Moreover, whole-mount *in situ* hybridization (WISH) assays further confirmed the induced transcriptional level of caspase 3 in pharyngeal region, eyes and optic tectum of *nup62l*-mutants (Figure 4C; Supplementary Figure S4C,D). 

**Figure 5:**
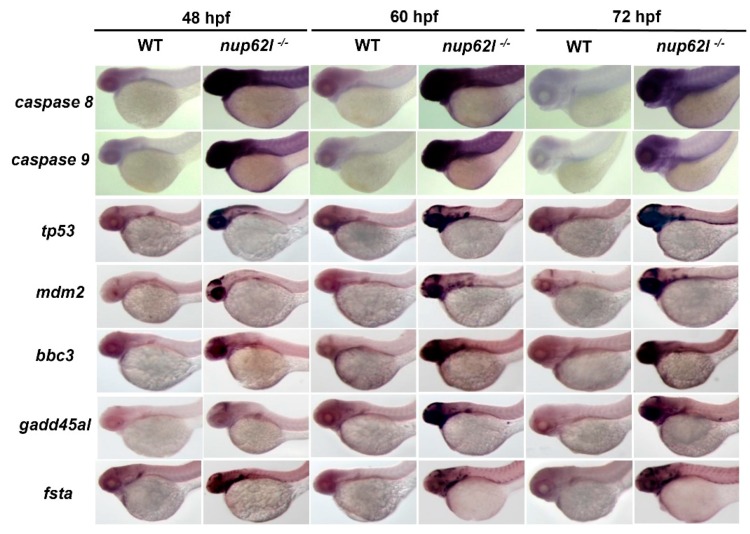
Loss of Nup62l activated intrinsic and extrinsic apoptotic pathways. WISH assays showing lateral views of the expression of various apoptosis-related genes (*caspase 8*, *caspase 9*, *tp53*/*p53*, *mdm2*, *bbc3*, *gadd45al* and *fsta*) in WT siblings and *nup62l*-mutants at different stages.

Next, we employed quantitative real-time PCR (qRT–PCR) to determine the expression of a set of well-known genes related to apoptosis at several key stages during embryogenesis. We found that a number of genes, including *caspase 8*, *caspase 9*, *tp53*/*p53*, *mdm2*, *bbc3*, and *fsta*, were highly up-regulated in *nup62l*-mutants at and after 48 hpf, while evidently increased levels of *gadd45al* and *fadd* were observed from 60 hpf onward (Supplementary Figure S5). WISH assays showed a general tendency similar to the qRT–PCR results, which is evidenced by up-regulated levels of *caspase 8*, *caspase 9*, *tp53*/*p53*, *mdm2*, *bbc3*, *gadd45al* and *fsta* in PA region, eyes and optic tectum of *nup62l*-mutants at examined stages (Figure 5; Supplementary Figure S6). These observations indicate that the inactivation of *nup62l* could trigger intrinsic and extrinsic apoptotic pathways in specific tissues such as the PA region of developing zebrafish.

### 3.4. Activation of the p53-Driven Apoptotic Pathway Contributed to the Impaired Formation of PA in Nup62l-Mutants

**Figure 6:**
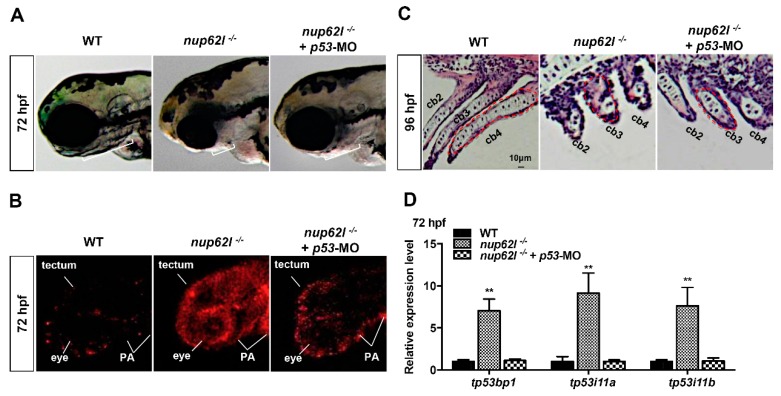
Activation of p53-dependent apoptotic pathway contributed to the defective formation of PA in *nup62l*-mutants. **(A)** Partial rescue of morphological defects in PA of *nup62l*-mutants injected with 5 ng *p53*-MO as shown by the lengths of white lines. (**B**) TUNEL assays were performed to assess the apoptosis in pharyngeal region of *nup62l*- and *p53*- double inactivated mutants compared to WT and *nup62l*-mutants at 72 hpf. Embryos were imaged by confocal microscopy. PA region, eyes and optic tectum were labeled. (**C**) PA sagittal sections at 96-hpf of WT, *nup62l*-mutants or *nup62l*-mutants injected with 5 ng *p53*-MO were analyzed with hematoxylin and eosin staining. The 2nd-4th ceratobranchials (cb) were labeled. (**D**) Expression levels of genes (*tp53bp1*, *tp53i11a* and *tp53i11b*) implicated in p53-dependent apoptotic pathway were analyzed by qRT–PCR in WT, *nup62l*-mutants or *nup62l*-mutants injected with 5 ng *p53*-MO at 72 hpf. Expression levels were normalized to WT embryos. Data expressed as Mean ± SD were representatives of three independent experiments. ** *p* < 0.01.

Tp53/p53 is a key signal molecule of both intrinsic and extrinsic apoptotic pathways, which can regulate the expression of genes hastening apoptosis and cell cycle arrest [54]. Abnormally elevated expression of *tp53*/*p53* in *nup62l*-mutants raised the possibility that p53-dependent apoptosis might be a key cause of the PA defects. To address whether *p53*-deficiency could suppress the increased apoptosis and PA defects, we knocked down *p53* in *nup62l*-mutants by the injection of *p53* morpholino (*p53*-MO) at the one-cell stage. As shown in Figure S7A, injection of *p53*-MO rescued the abnormally enhanced expression of *tp53* downstream target genes *tp53i11a/b* in a dose-dependent manner in *nup62l*-mutants and could restore the enhanced expression to the WT level at the dosage of 5 ng/embryo. Thus, we selected a dosage of *p53*-MO at 5 ng/embryo for the morphological observation and TUNEL assays at 72 hpf. We found that morphological PA defects of *nup62l*-mutants could be partially rescued by *p53*-MO injection (Figure 6A; Supplementary Figure S7B). TUNEL assays showed that the number of TUNEL-positive cells in *nup62l*- and *p53*-double deficient embryos was obviously reduced in PA, eyes and optic tectum when compared to that in *nup62l*-mutants but was still slightly more than that in WT siblings (Figure 6B; Supplementary Figure S7C,D). Similarly, the disorganized and shrunk chondrocytes in sagittal sections of *nup62l*-mutant arches were partially recovered by injection of *p53*-MO (Figure 6C; Supplementary Figure S7E). These data suggest an insufficient amount of p53 could mainly rescue the PA defects and apoptosis in *nup62l*-mutants. 

In humans, *TP53BP1* (p53-binding protein 1) and *TP53I11* (p53 inducible protein 11) are closely associated with the p53-mediated apoptotic pathway. Therefore, we detected the expression levels of *tp53*, *tp53bp1* and *tp53i11a/b* in zebrafish. As expected, qRT–PCR results revealed increased expression levels of these genes in p53-driven apoptotic pathway in *nup62l*-mutants when compared to those in WT controls (Figure 6D; Supplementary Figure S7F), and the enhanced expression of *tp53bp1* and *tp53i11a/b* were rescued by p53 knockdown (Figure 6D). Thus, the abnormal activation of the p53-dependent apoptotic pathway is involved in apoptosis in PA and the loss of pharyngeal cartilage in *nup62l*-mutants. 

### 3.5. Extensive Apoptosis Occurring in PA of Nup62l-Mutants is Attributable to Inactivation of Wnt/β-Catenin Signaling

**Figure 7:**
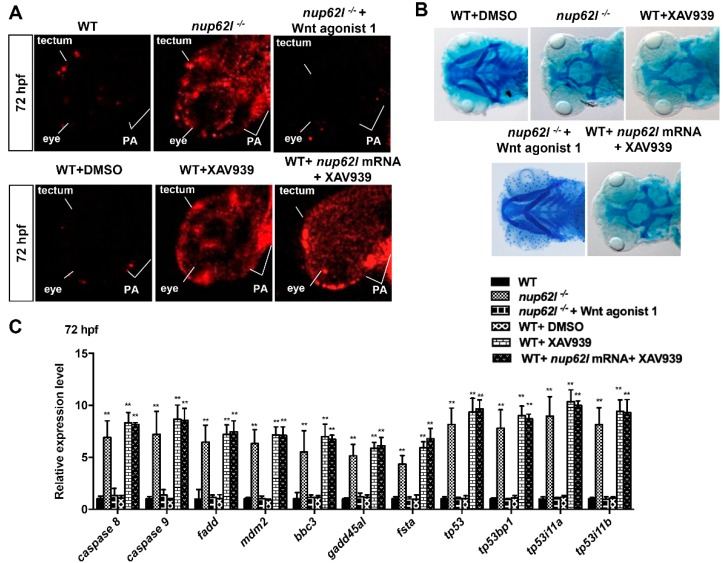
Suppression of Wnt/β-catenin signaling by Nup62l deprivation activated multiple apoptotic pathways. (**A**) Apoptotic cells were detected by TUNEL assays at 72 hpf in PA of WT embryos, *nup62l*-mutants, *nup62l*-mutants treated with 10 μM Wnt agonist 1, WT treated with 0.15% DMSO or 15 μM XAV939, and WT treated with both 300 ng *nup62l* mRNA and 15 μM XAV939. A significantly increased apoptosis was observed in *nup62l*-mutants and WT embryos treated with XAV939. (**B**) Alcian blue staining at 96 hpf of *nup62l*-mutants treated with or without 10 μM Wnt agonist 1, WT treated with 0.15% DMSO or 15 μM XAV939, and WT treated with both 300 ng *nup62l* mRNA and 15 μM XAV939. Note a greatly decreased cartilages in PA of *nup62l*-mutants and larvae treated with 15 μM XAV939. Images are ventral views with anterior to the left. (**C**) Relative mRNA levels of genes for apoptotic pathways. Embryos treated as indicated above were collected at 72 hpf for qRT–PCR assays. Data expressed as Mean ± SD were representatives of three independent experiments. ** *p* < 0.01.

Wnt signaling is an evolutionarily conserved pathway that plays key roles in many aspects of embryonic development and a variety of human disorders [55]. An increasing number of compelling evidence has shown that Wnt/β-catenin signaling participates in the regulation of cell apoptosis [24,56]. Moreover, we have previously demonstrated that Nup62l up-regulates the activity of Wnt/β-catenin pathway through interacting with and facilitating nuclear import of β-catenin-1/2 in zebrafish [37]. Thus, it is possible that the activation of multiple apoptotic pathways in *nup62l*-mutants might be caused by the inactivation of Wnt/β-catenin signaling. To address this question, WT siblings were treated with XAV939, a small molecule drug to inhibit β-catenin-mediated transcription [57]. WT embryos at 6 hpf were treated with either 15 μM XAV939 or 0.15% DMSO (as a vehicle control) and processed for TUNEL analysis at 72 hpf. In DMSO-treated WT controls, only faint TUNEL-positive signals appeared in PA, eyes and optic tectum; however, WT embryos that were exposed to 15 μM XAV939 to inhibit Wnt/β-catenin signaling showed a drastic induction of apoptotic cells in these tissues, which are similar to those in *nup62l*-mutants (Figure 7A; Supplementary Figure S8A,B). As expected, about 89% of embryos that were treated with XAV939 and stained with alcian blue exhibited a total loss of cartilages in seven PA at 96 hpf (Figure 7B; Supplementary Figure S8C–E). In turn, we treated *nup62l*-mutant embryos with a Wnt activator (Wnt agonist 1), which functions through induction of β-catenin-dependent transcriptional activity [58], and found that activation of Wnt/β-catenin signaling could rescue the aberrant apoptosis and impaired PA caused by loss of Nup62l (Figure 7A,B; Supplementary Figure S8). We further evaluated the expression of apoptosis-specific genes using qRT–PCR. XAV939-treated WT embryos resembled *nup62l*-mutants to express high levels of *caspase 8*, *caspase 9*, *fadd*, *tp53*/*p53*, *mdm2*, *bbc3*, *gadd45al* and *fsta* at 72 hpf. Importantly, a highly increased expression of p53-apoptosis related genes *tp53bp1* and *tp53i11a/b* in *nup62l*-mutants was also detected in XAV939-treated WT embryos (Figure 7C). In turn, treatment with Wnt agonist 1 could rescue the increased expression of these apoptotic marker genes including p53-mediated apoptosis genes (*tp53*, *tp53bp1* and *tp53i11a/b*) in *nup62l*-mutant embryos (Figure 7C).

Taken together, these results indicate that the loss of Nup62l activated intrinsic and extrinsic apoptotic pathways through suppression of Wnt/β-catenin signaling in Nup62l-expressing tissues including the PA region of zebrafish.

## 4. Discussion

A few studies have proposed the implications of nucleoporins in PA formation [32,59], but functions and mechanisms of nucleoporins during the morphogenesis of craniofacial cartilages in vertebrates remain to be well explored. In this study, we demonstrated the pivotal roles of zebrafish Nup62l in the development of PA cartilages. Using the CRISPR/Cas9 technology, we generated a zebrafish *nup62l*-mutant line. We found that the loss-of-function of *nup62l* led to a morphogenetic malformation in PA due to the developmental failure of PA cartilages. In comparison with WT larvae, homozygous *nup62l*-mutants exhibited dysplastic PA as characterized by a thinned and shortened pharyngeal region. The pharyngeal region affected by loss of Nup62l exactly matches the position that *nup62l* RNA was predominantly expressed during zebrafish development [37]. Interestingly, the phenotype of *nup62l*-mutants largely resembles that of zebrafish *nup107*-mutants that is attributed to the impaired nuclear pore function [32]. Moreover, the development of chondrocytes was severely impaired by Nup62l deprivation since all of seven PA in *nup62l*-mutants failed to be stained with alcian blue. Therefore, the tissue-specific expression of *nup62l* in pharyngeal region is essential for the appropriate development of PA.

The formation of zebrafish cartilage involves two distinctive developmental phases. The first phase forms rapidly the cartilage morphogenesis accompanying chondrocyte differentiation during 48 to 72 hpf [6]. The following extended phase of rapid cartilage growth occurs through chondrocytes divisions in the early feeding and swimming fish from 72 hpf onwards [60]. We investigated the stages of pharyngeal cartilage formation at which Nup62l is required. During the dynamic process, Nup62l is detectable in the pharyngeal region at and after 48 hpf [37]. Loss of Nup62l did not impair the specification and migration of CNCCs, as characterized by the unperturbed expression of early crest markers *sox9a*, *sox10* and *foxd3* as well as postmigratory neural crest marker *dlx2a*. However, Nup62l played indispensable roles in prechondrogenic condensation and differentiation of pharyngeal chondrogenic progenitors as evidenced by restricted expression of corresponding marker genes *sox9a* and *col2a1a* in *nup62l*-mutants. Thus, our findings suggest the involvement of Nup62l in pharyngeal cartilage morphogenesis.

The Nup62 complex consists of Nup62, Nup58, Nup54, and Nup45, which lines the central channel of the NPC [61]. Previous studies have shown that Nup62 acts as a crucial controller of the nucleocytoplasmic transport events [34] through direct interaction with several nuclear transport factors, including importin-β and NTF2 [62]. Our previous report [37], and this study, have unraveled that Nup62l plays critical roles in zebrafish morphogenetic processes. These findings raise the question of how the loss of Nup62l impeded the formation of cartilages in PA. In this study, we noticed that *nup62l*-knockout led to an obviously reduced number of chondrocytes in the sagittal sections, implying that the absence of PA in *nup62l*-mutants may be caused by an increase of cell death. Indeed, we found that the loss of Nup62l resulted in severe apoptosis within Nup62l-expressing tissues, specifically in the defective PA. This observation was further supported by significantly elevated expression of many genes associated with intrinsic and extrinsic apoptotic pathways in PA, eyes and optic tectum. Moreover, the p53-dependent apoptotic signaling is involved in the formation of PA cartilage as evidenced by elevated expression level of *tp53*/*p53* and a partial rescue of PA failure by *p53*-MO in *nup62l*-mutants. 

It has been well documented that Wnt signaling functions in the regulatory of apoptosis [63]. For instance, Wnt signaling modulates apoptosis during both development and injury in various cell populations of neurons, cardiomyocytes, vascular smooth muscle cells and endothelial cells [64]. The regulation of apoptosis by Wnt signaling can be mediated through multiple mechanisms, such as SFRP2 expression [65], Wnt-BMP signaling loop [65], GSK-3β-NF-kB signaling [66], Sox10 [67], and Wnt/β-catenin signaling [24]. Antagonizing the activity of Wnt/β-catenin pathway triggers apoptosis since genetic mutation of β-catenin results in apoptotic loss of craniofacial structures, midbrain, cerebellum and sensory neurons in mice [24]. Moreover, repression of Wnt/β-catenin activity induces apoptosis in some carcinoma cells [68] and activation of Wnt/β-catenin pathway restrains chemotherapy-induced apoptosis [69]. In this study, we found that a number of intrinsic and extrinsic apoptotic pathways were triggered in the PA, eyes, and optic tectum of WT embryos treated with Wnt/β-catenin inhibitor XAV939, while activation of Wnt/β-catenin signaling by Wnt agonist 1 could rescue the increased apoptosis in *nup62l*-mutants. These findings indicated that loss of Nup62l led to aberrant activation of multiple apoptotic pathways through inactivation of Wnt/β-catenin signaling in *nup62l*-mutants. However, the overexpression of *nup62l* mRNA could not rescue the apoptosis and phenotype that were induced by XAV939 (Figure 7; Supplementary Figure S8). We have previously demonstrated that Nup62l functions to activate Wnt/β-catenin signaling through interacting with and facilitating of nuclear import of β-catenin-1/2 [37], so we speculate that overexpression of *nup62l* can facilitate the accumulation of β-catenin-1/2 in nucleus, but not rescue the disrupted effect of XAV939 on β-catenin-mediated transcription. 

**Figure 8:**
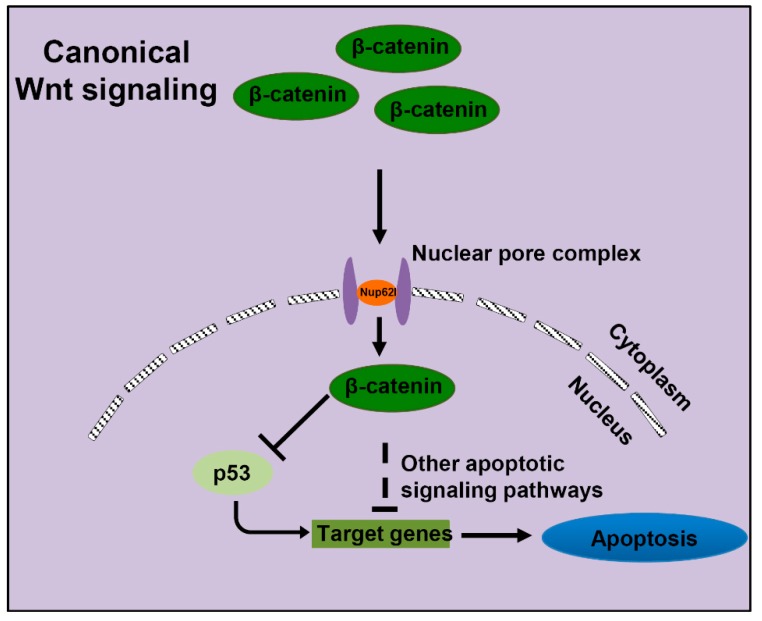
A schematic model for the connection between Nup62l-regulated Wnt/β-catenin and apoptotic signaling pathways.

Indeed, a series of pro-apoptotic genes, such as *PTEN*, *NFKBIA*, *FADD*, *TP53BP1* and *TP53I11*, are dramatically up-regulated in β-catenin-knockdown HeLa cells and these genes belong to several critical apoptotic pathways, including PTEN-PI3K-AKT, NF-κB and p53 apoptotic pathway [70]. Here, we detected the activation of p53 apoptotic pathway as supported by obviously elevated expression of corresponding genes *tp53*, *tp53bp1* and *tp53i11a/b* after restricting the activity of Wnt/β-catenin signaling in zebrafish. It has been shown that *TP53BP1*-encoded 53BP1 can interact with p53 to facilitate p53-mediated transcriptional activation [71] and the p53 direct target gene *TP53I11* can function to promote cell apoptosis and contribute to chemosensitivity of cells to arsenic trioxide [72]. Therefore, inhibition of Wnt/β-catenin pathway by Nup62l depletion may trigger p53-dependent apoptosis through up-regulation of pro-apoptotic genes *tp53bp1* and *tp53i11a/b*. The normal activation of Wnt/β-catenin signaling is essential for PA development in zebrafish. In this study, we outlined a novel connection between Nup62l-regulated Wnt/β-catenin and apoptotic signaling pathways. Under normal conditions, zebrafish Nup62l facilitates the activation of Wnt/β-catenin signaling and thus ensures the proper control of apoptotic signaling pathways including p53-dependent apoptosis associated PA development (Figure 8). As key regulators of nucleocytoplasmic communication, nucleoporins have been shown to autonomously play developmental roles in different cell types. Depletion of Nup62, Nup214 or Nup88 in cyst cells led to cell-autonomous defects in mRNA export in Drosophila [73]. The loss of Nup98-96 caused dramatic cell-autonomous effects in male germ cells of Drosophila, thus leading to premature differentiation of these cells to spermatocytes [74]. Moreover, Nic96p [75] and Nup153p [76], which contain sequences that can function autonomously as nuclear localization signals, are essential for cell viability. Thus, it is likely that Nup62l functions autonomously in cells during PA development through regulation of Wnt/β-catenin signaling. 

In addition to an essential role of apoptosis in tissue homeostasis and morphogenesis of vertebrates, abnormal regulation of apoptosis has important implications in a wide range of developmental abnormalities and human diseases. For instance, excessive apoptosis is thought to lead to neurodegenerative and neuromuscular diseases as characterized by the progressive loss of neurons, while disruption of normal apoptosis can lead to cancers where too little apoptosis occurs [77]. The dysregulation of Wnt/β-catenin pathway in adult organism is involved in multiple types of oncogenesis and neurodegenerative disorders [78,79]. Thus, a further dissection of the precise molecular mechanisms underlying the control of apoptosis by the Wnt/β-catenin pathway may provide salutary therapeutic avenues for cancers and neurodegenerative diseases. Moreover, the regulatory effects of Nup62l and Wnt/β-catenin signaling on PA formation indicate that Nup62l and Wnt/β-catenin would be attractive therapeutic targets for treatment of craniofacial syndromes. The development of novel nanoparticles for the effective and safe delivery of small molecule drugs into target cells will finally facilitate the treatment of apoptosis-associated human diseases.

## Figures and Tables

**Table 1:** Primers used in this study.

Primer Names	Primer Sequences (5’–3’)
sox9a-F	CAG AGC GCA GTA CGA CTA TT
sox9a-R	GTA GCT GAA GGT GGA GTA CAG
col2a1a-F	CTG GTG ATC GTG GTG AGA TTG
col2a1a-R	TCA CCC TGC TCT CCC TTA TT
sox9b-F	GGC GCT CCT GCT AAC AAT AA
sox9b-R	ACC CTA ACC CTA ACC CTA ACC
caspase 8-F	GGA GAG AGA AAG GAG GAG AAA C
caspase 8-R	CCG CTG GGT CAG TAT GTA AT
bbc3-F	GGT TTC AAG CAC TTC CCT TAG A
bbc3-R	CCG ACG CAA ACA CAG AAA TG
fadd-F	AGT CGG TCA GAC AGT TCT TAT TG
fadd-R	GTG TTG ATT CTC TCT CGC TCT T
fsta-F	TGT GCC AAA CAG CAC ATT ATT C
fsta-R	CAG GAC CAC AGT CCA CAT TAT C
gadd45al -F	GAC GGA AGC TCC TTC AGA ATA C
gadd45al -R	GTC CTC AGA AAG TCC CAC AAA
mdm2-F	AAC AGC AAC TCG GAT GTA GG
mdm2-R	CCA CCT CAA ACT CCA CAC TAA
caspase 9-F	GGA GGA GGT GAG AAG GAT ATT G
caspase 9-R	CTG CTA GAG GAC ATG GGA ATA G
tp53-F	GTA CAA GTC CCT CCT GGA AAT C
tp53-R	GGC AAA TGC GTG TAA ACA GTA A
tp53bp1-F	CTA ACC CTG TCG CAT CCT TAT G
tp53bp1-R	AGG CTG AGA GTC CTC AAC TAT
tp53i11a-F	GAC CTC AGT AGA TGG TGG AAT G
tp53i11a-R	GAG ATG GAC GCA GAA CTC AA
tp53i11b-F	AGG AGA GGA TGA TGA TGG AGA G
tp53i11b-R	GGC GGA GAA GAG AAT CCA TAA C

## Supplementary Materials

The following are available online at https://www.mdpi.com/2073-4409/8/9/1038/s1, Figures S1–S8.
