# Supplementary material for: Nucleoporin 62-Like Protein Is Required for the Development of Pharyngeal Arches through Regulation of Wnt/β-Catenin Signaling and Apoptotic Homeostasis in Zebrafish
Source: Cells. 2019 Sep 5;8(9):1038. doi: 10.3390/cells8091038 (PMC6770318; doi:10.3390/cells8091038)
Supplement: Supplementary file 1 [file cells-08-01038-s001.pdf]

SUPPLEMENTARY MATERIALS

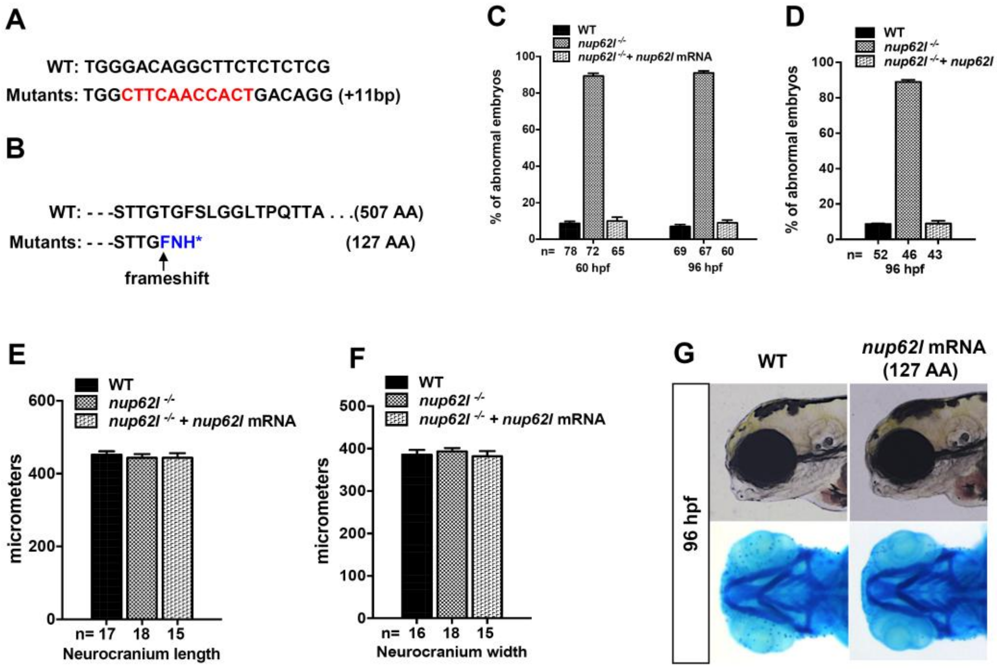

Figure S1. (**A**) The 11- nucleotide insertion sequences in *nup62l*-mutants were shown in red. (**B**) An alignment of the Nup62l sequences encoded in WT and *nup62l*-mutants. The 11-nucleotide insertion caused a frame-shift of the open reading frame for Nup62l, leading to a premature stop codon (marked as *) in *nup62l*-mutants. (**C**, **D**) The percentage of defective embryos as shown in Figure 1C and Figure 1D, respectively. (**E, F**) Cartilage measurements of the length and width of neurocranium in WT, *nup62l*-mutants or *nup62l*-mutants injected with 300 pg *nup62l* mRNA. Data were representatives of three independent experiments. The total numbers detected (n) were shown at the bottom. (**G**) The 96-hpf PA phenotype of WT siblings or WT injected with 300 pg N-terminal 381bp (127AA) partial mRNA of *nup62l* coding sequences.

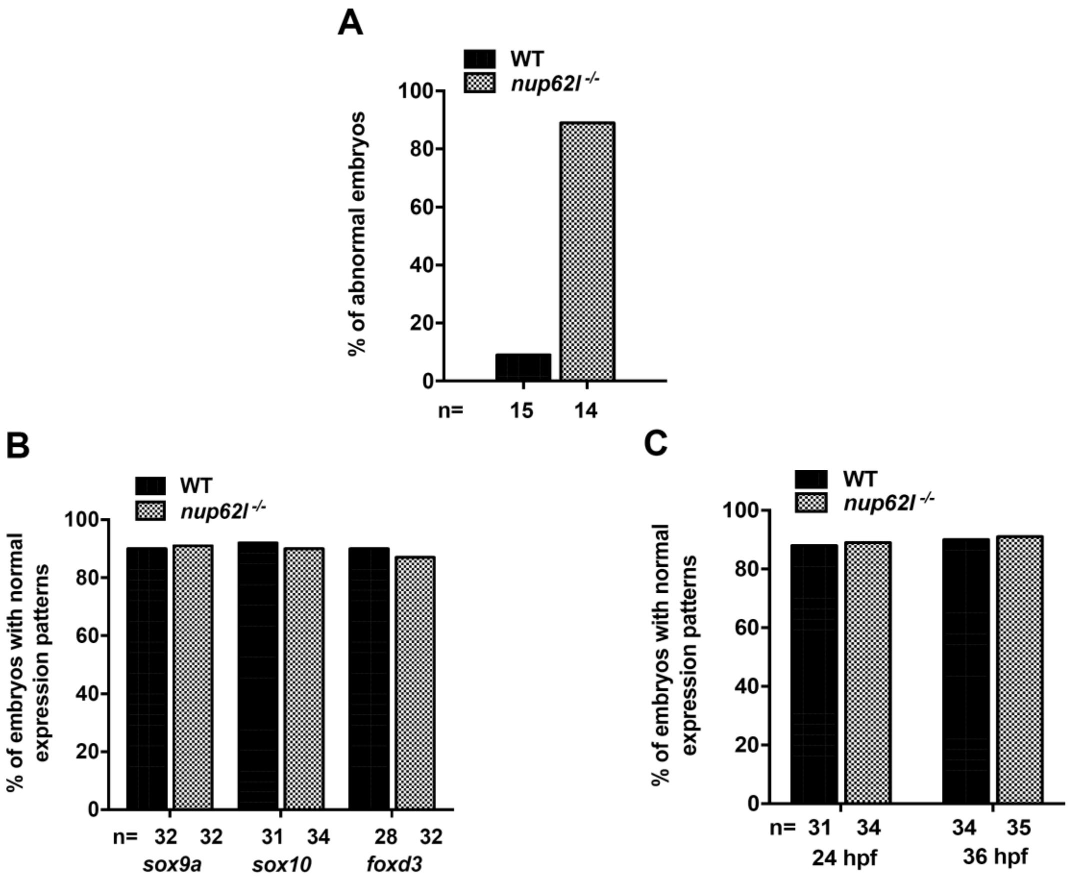

Figure S2. (**A**) The percentage of defective embryos as displayed in Figure 2A. (**B**, **C**) The percentage of embryos with representative expression patterns shown in Figure 2B and 2C, respectively. The number of embryos (n) and developing stages examined were indicated below the bars.

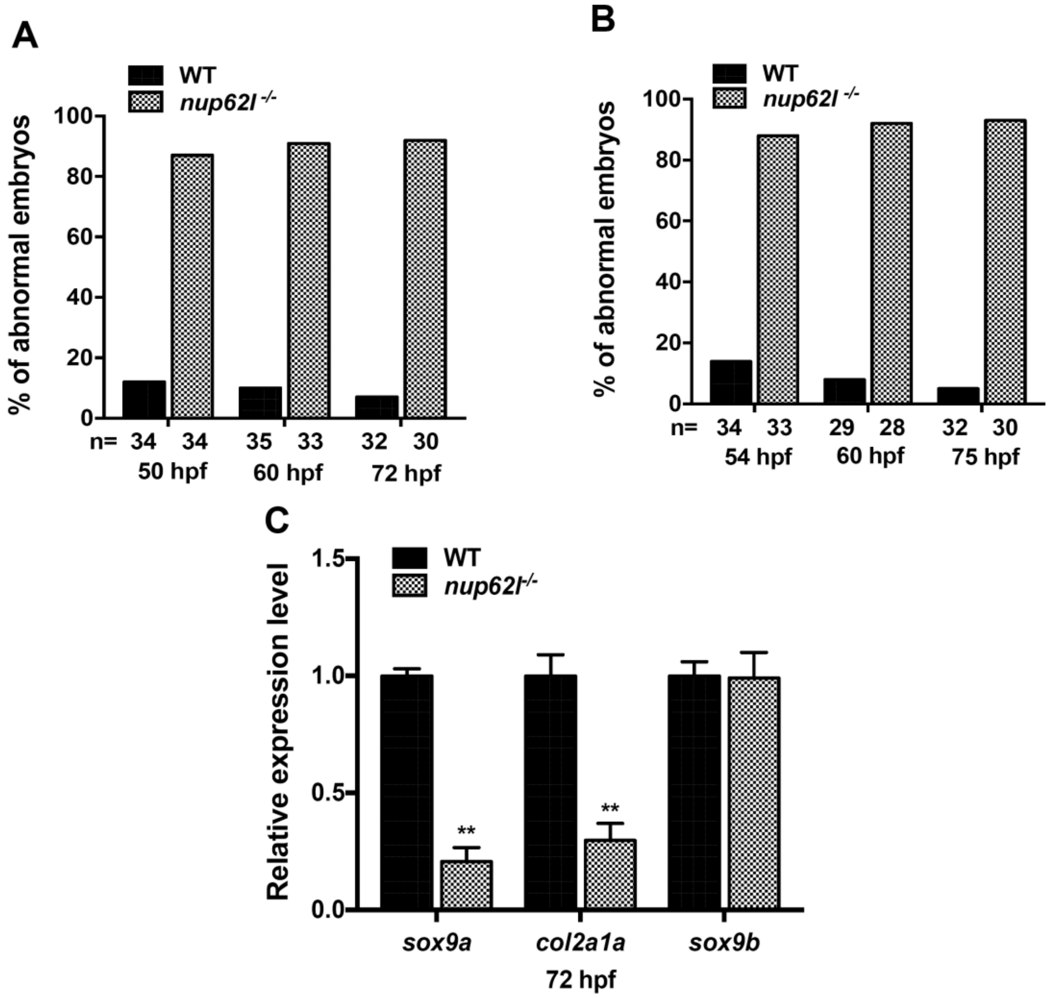

Figure S3. The percentage of *nup62l*-mutants or WT siblings displaying impaired expression patterns of genes *sox9a* (**A**) and *col2a1a* (**B**) as shown in Figure 3. (**C**) The mRNA levels of genes *sox9a*, *sox9b* and *col2a1a* at 72 hpf. Expression levels were normalized to WT. The data expressed as Mean ± SD were representatives of three independent experiments containing 50 embryos per sample. **, $p<0.01$.

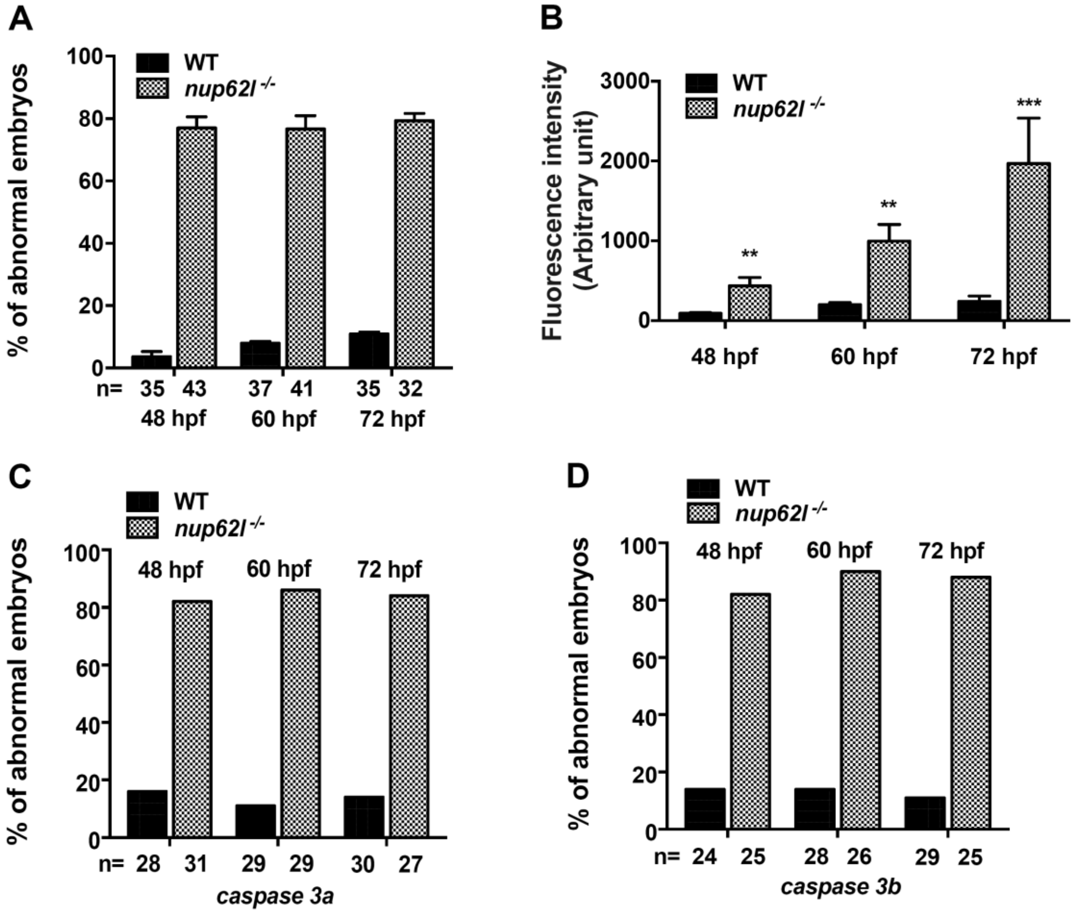

Figure S4. (**A**) Quantification of *nup62l*-mutants showing increased apoptosis in PA at 48 hpf, 60 hpf or 72 hpf as demonstrated in Figure 4A. (**B**) Immunofluorescence intensities of approximately 20 WT or *nup62l*$^{-/-}$ embryos were determined using the ImageJ software (National Institutes of Health, Bethesda, MD) and expressed as arbitrary units. Values are expressed as Mean ± SD from three independent experiments. **, p<0.01; ***, p<0.001. (**C**, **D**) WT or *nup62l*-mutants were quantified by abnormal expression of *caspase 3a* and *caspase 3b* at the indicated stages in Figure 4C. The data were representatives of three independent experiments.

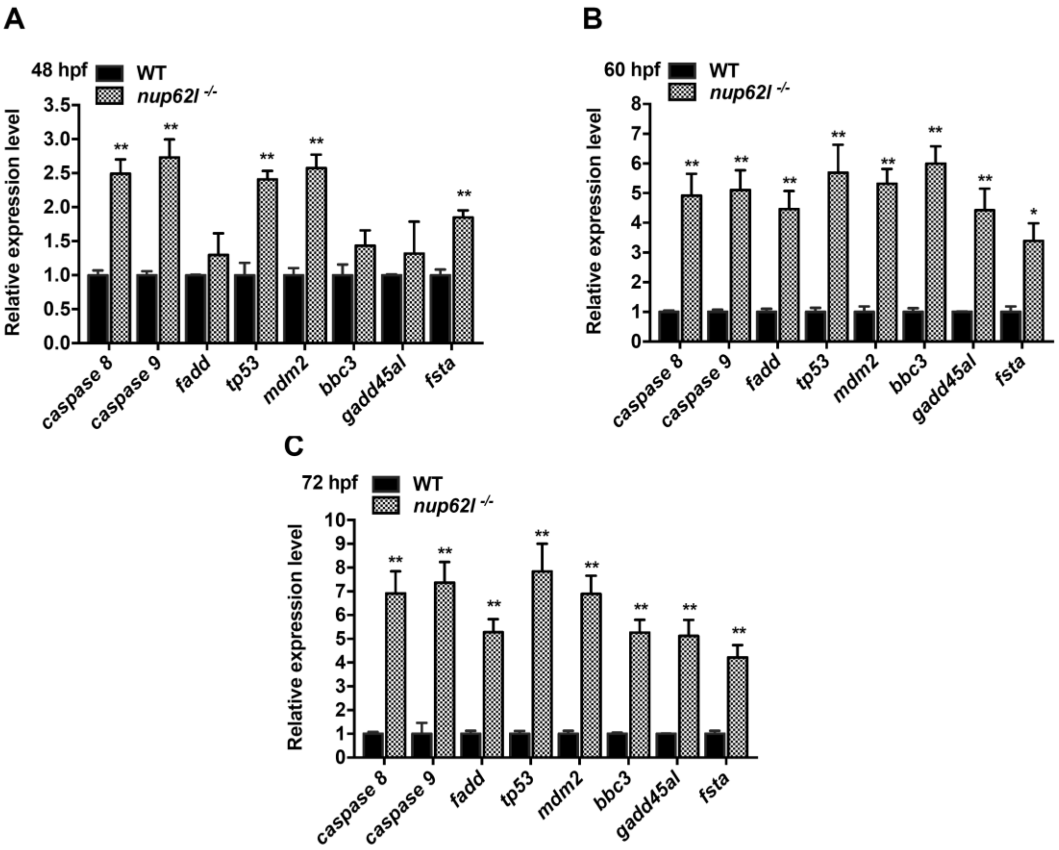

Figure S5. (**A**-**C**) The mRNA levels of genes involved in intrinsic and extrinsic apoptotic pathways were analyzed with qRT–PCR. Total mRNAs were extracted from WT or *nup62l*-mutants at 48 hpf, 60 hpf or 72 hpf. Expression levels were normalized to WT. The data expressed as Mean ± SD were representatives of three independent experiments containing 50 embryos per sample. *, $p<0.05$; **, $p<0.01$.

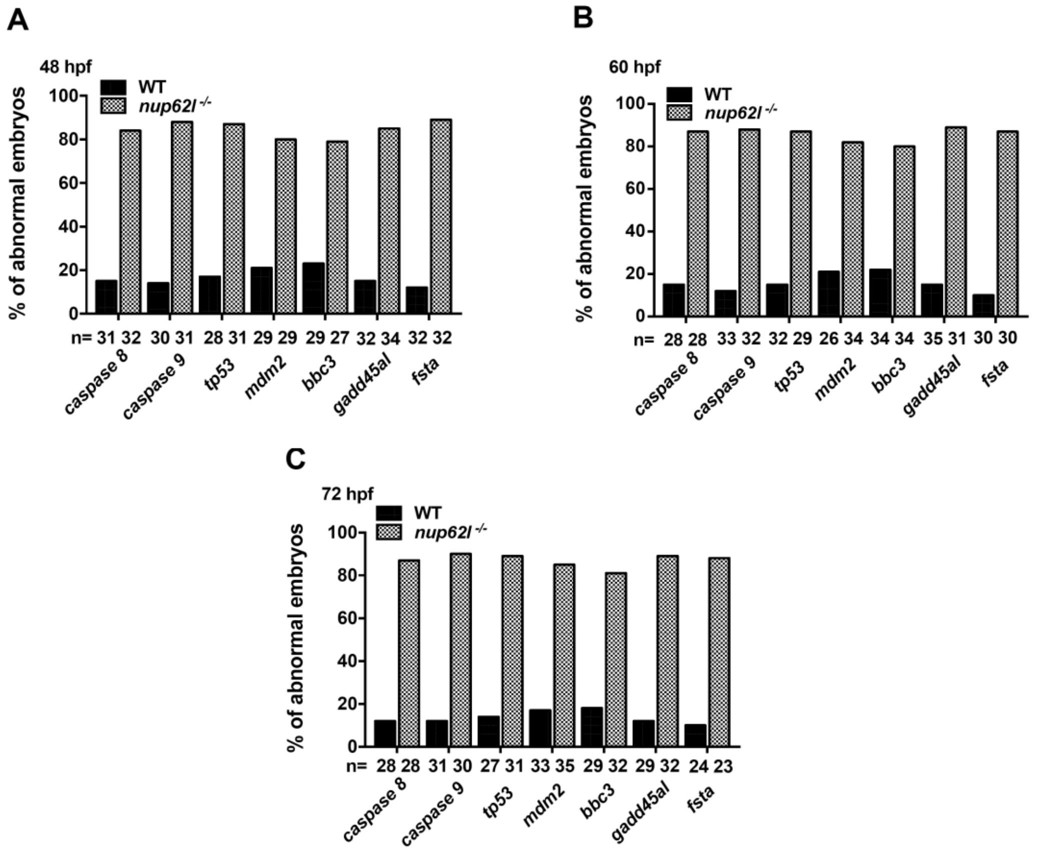

Figure S6. (**A-C**) The percentage of *nup62l*-mutants carrying affected patterns of WISH assays for apoptosis-related genes as shown in Figure 5. The total numbers of embryos detected were indicated at the bottom of the bars.

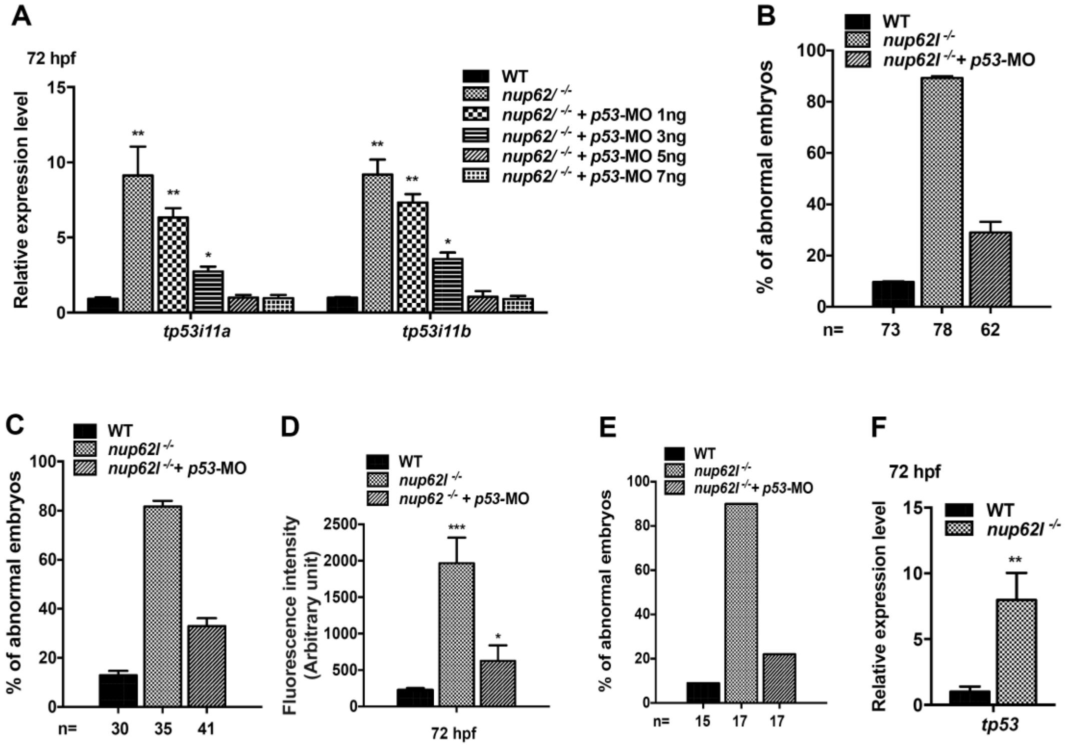

Figure S7. (**A**) The mRNA expression levels of *tp53i11a* and *tp53i11b* in WT or *nup62l*$^{-/-}$ embryos injected with or without 1, 3, 5 or 7 ng *p53*-MO. (**B**) Quantification of embryos showing abnormal pharyngeal morphology indicated in Figure 6A. (**C**) The ratios of embryos showing the abnormal apoptosis in pharyngeal regions at 72 hpf as shown in Figure 6B. (**D**) Immunofluorescence intensities of approximately 20 WT, or *nup62l*$^{-/-}$ embryos injected without or with 5 ng *p53*-MO were expressed as arbitrary units. (**E**) The ratios of 96-hpf WT, *nup62l*-mutants or *nup62l*-mutants injected with 5 ng *p53*-MO and showing aberrant chondrocytes of PA in Figure 6C. (**F**) The mRNA expression levels of *tp53* in WT or *nup62l*$^{-/-}$ embryos at 72 hpf. Values are expressed as Mean ± SD from three independent experiments. *, p<0.05; **, p<0.01; ***, p<0.001.

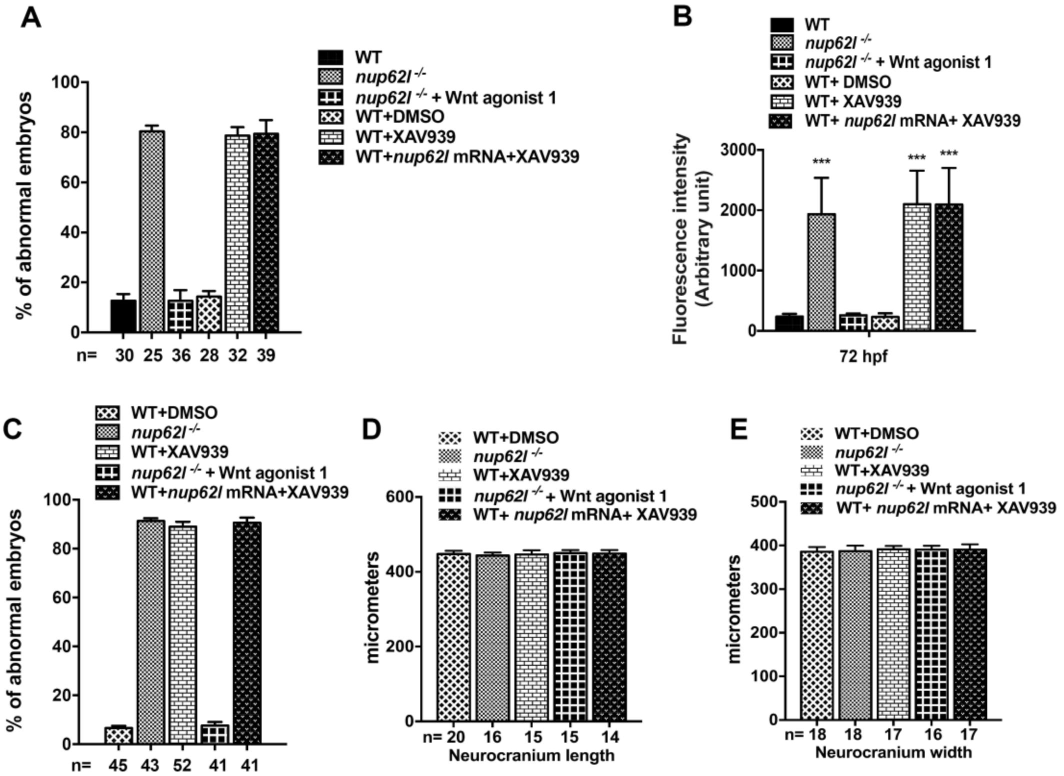

Figure S8. Embryos showng the aberrant apoptosis levels as imaged in Figure 7A, were quantified in (**A**). (**B**) Immunofluorescence intensities of approximately 20 embryos showing in Figure 7A were expressed as arbitrary units. Values are expressed as Mean ± SD from three independent experiments. ***, p<0.001. (**C**) Statistical results of embryos showing defective pharyngeal cartilage patterns in Figure 7B. (**D**, **E**) Cartilage measurements of the length and width of neurocranium in embryos showing in Figure 7B. The total numbers of embryos examined were displaced below the bars.
